# Intradiscal pharmacokinetics of oral antibiotics to treat Chronic Lower Back Pain

**DOI:** 10.1038/s44259-023-00002-7

**Published:** 2023-05-10

**Authors:** Lloyd G. Czaplewski, Marcus Zeitlinger, Joseph F. Standing

**Affiliations:** 1 Persica Pharmaceuticals Ltd, 7 Denne Hill Business Centre, Womenswold, Canterbury, Kent, CT4 6HD UK; 2 Medical University of Vienna, Department of Clinical Pharmacology, Clinical Pharmacokinetics / Pharmacogenetics and Imaging, Waehringer Guertel 18-20, 1090 Vienna, Austria; 3 Infection, Immunity & Inflammation Department, University College London, Great Ormond Street Institute of Child Health, 30 Guilford St, London, WC1N 1EH UK

**Keywords:** Antibiotics, Pharmacokinetics, Pharmacodynamics, Clinical pharmacology

## Abstract

Oral coamoxiclav and amoxicillin, for extended dose regimens of up to 100 days, have shown benefit in the treatment of Chronic Lower Back Pain (CLBP) associated with vertebral bone oedema, known as Modic type 1 changes, which may be caused by a bacterial infection, but the magnitude of clinical improvement has been variable. The objectives of this review were to use sparse data from the literature to estimate the exposure of amoxicillin in the intervertebral disc, and to determine whether adequate antimicrobial exposure may have been achieved. Exposure to amoxicillin in herniated disc tissue was approximately 6.5% of the serum concentration. Dosing of oral amoxicillin, Q12h, at doses of up to 1,000 mg is unlikely to lead to effective exposure in disc tissue. Mean exposure to 500 mg or 750 mg of oral Q8h amoxicillin may reach the efficacy target for ~50% of *Cutibacterium acnes* strains, but not for 90% of *C. acnes* strains. Mean exposure to 1,000 mg of oral amoxicillin Q8h may reach the target exposure for 90% of strains. Oral amoxicillin CLBP studies may all be underdosed. More than 1400 patients with CLBP and Modic type 1 changes have been exposed to oral amoxicillin for up to 100 days, with no apparent evaluation of systemic or intradiscal pharmacokinetics. Additional clinical evaluations of amoxicillin and alternative antibiotics, their dose regimens, and intradiscal pharmacokinetics are warranted to optimize treatment for this indication. Expertise in antibacterial pharmacokinetics and pharmacodynamics should be included in the design and execution of future studies.

## Introduction

Chronic Lower Back Pain (CLBP) associated with vertebral bone oedema, known as Modic type 1 changes, is thought to be caused or aggravated by a putative bacterial infection. Oral co-amoxiclav and amoxicillin for durations of up to 100 days have been demonstrated to treat CLBP successfully. However, the magnitude of clinical improvement in studies is variable, possibly due to differences in the dosage regimens used^1^. Studies using quantitative microbiology, immunohistology, proteomics and animal models of disc infection which recapitulate human disc degeneration and development of vertebral bone oedema, unequivocally demonstrate potential for a bacterial aetiology of CLBP, focusing on *C. acnes* as the most likely cause^1^. CLBP is one of the leading causes of disability and treatment options are limited^1–3^. Comprehensive evaluation and optimization of antimicrobial chemotherapy to treat a subset of patients with CLBP associated with putative bacterial disc infection could provide substantial patient benefits.

Clinical studies evaluating antibiotics to treat CLBP associated with Modic changes type 1, which are bone marrow lesions seen within a vertebral body on magnetic resonance imaging^4^, or spinal disc herniation, caused or aggravated by a putative bacterial infection, have focused on the use of coamoxiclav (amoxicillin clavulanate), with oral doses of amoxicillin 500 mg, 750 mg, and 1000 mg three times a day (Q8h) or 500 mg two times a day (Q12h) for an extended period of up to 100 days (Table 1)^5–13^.

**Table 1:** Summary of clinical trials that evaluated oral antibiotics to treat nonspecific chronic low back pain or herniation.

Publication et al.	Year	Study Design	Subject characteristics	Antibiotic	Dose of antibiotic	Duration (Days)	Timepoint(s) for efficacy assessment	Effect on LBP NRS	Effect on disability RMDQ
Albert^5^	2008	Uncontrolled open label	CLBP with prior herniation and Modic Type 1 changes. Failed conservative treatment *N* = 29	Amoxicillin clavulanate (Spektramox™)	0.5 g Q8h	90	90 days and 12 months	4 points (44%) median reduction at 90 days and 12 months	4 points (50%) median reduction at 90 days and 3 points (37.5%) reduction at 12 months
Albert^6^	2013	RCT	CLBP with prior herniation and Modic Type 1 changes. Failed conservative or surgical treatment *N* = 72 placebo *N* = 45 0.5 g Q8h *N* = 45 1.0 g Q8h	Amoxicillin clavulanate (Bioclavid™)	0.5 g Q8h 1.0 g Q8h The results for both antibiotic dose groups were pooled	100	90 days and 12 months	1.3 point (20.6%) median reduction versus placebo at 100 d and 2.6 point (41.3%) reduction at 12 months	2.5 point (17.8%) median reduction vs. placebo at 100 d and 7 point reduction (50%) at 12 months
Al Falahi^7^	2014	RCT	CLBP with prior herniation and Modic Type 1 changes. Failed conservative or surgical treatment *N* = 29 placebo *N* = 42 0.5 g Q12h	Amoxicillin clavulanate (Gloclav™)	0.5 g Q12h	100	100 days	1.2 point (20%) mean reduction vs. placebo at 100 d	2.8 point (18.9%) mean reduction vs. placebo at 100 d
Manniche^8^	2016	Uncontrolled open label	CLBP with prior herniation and Modic Type 1 changes. Failed conservative treatment *N* = 70 0.5 g Q8h 1 month, 1 g Q8h 2 months *N* = 77 0.5 g Q12h 3 months	Amoxicillin clavulanate (Product name not stated)	0.5 g Q8h 1 month, 1 g Q8h 2 months 0.5 g Q12h 3 months	90	6 months	53% responded to treatment with a ~30% mean reduction in pain. The non-responders reported a 2.1% mean reduction in pain. No differences in dose response were observed.	Not assessed
Palazzo^9^	2017	Uncontrolled open label	CLBP with Modic Type 1 changes. Failed conservative treatment *N* = 28	Amoxicillin clavulanate (Product name not stated)	0.5 g Q8h	100	100 days Some evaluation at 12 months	1.1 point (18.3%) mean reduction from baseline at 100 days	Not assessed
Gupta^10^	2017	Uncontrolled open label	CLBP with prior herniation and Modic Type 1 changes. Failed conservative treatment *N* = 11	Amoxicillin clavulanate (Product name not stated)	0.5 g Q8h	90	6 and 12 months	Not possible to summarise this.	Not assessed
Albert^11^	2017	Uncontrolled open label	CLBP with prior herniation and Modic Type 1 or Type 2 or mixed changes. Failed conservative or surgical treatment *N* = 987 at 100 days *N* = 602 at 12 months *N* = 270 at 24 months	Amoxicillin clavulanate (Bioclavid™)	1.0 g Q8h	100	12 and 24 months	2.0 point (33.3%) reduction versus placebo at 100 d, 2.7 point (45.0%) reduction at 12 months and 3.0 point (50%) reduction at 24 months	3.8 point (24.5%) reduction versus placebo at 100 d, 7.2 point (46.4%) reduction at 12 months and 8.1 point (52.2%) reduction at 24 months
Braten^12^	2019	RCT	CLBP with prior herniation and Modic Type 1 or Type 2 or mixed changes. Failed conservative treatment *N* = 56 placebo *N* = 55 Amoxicillin	Amoxicillin (Product name not stated) Tablets were also encapsulated to match placebo. Effect of encapsulation on bioequivalence not known	0.75 g Q8h	3 months	12 months	MC1 patients 0.7 point (13.4%) mean reduction versus placebo at 12 months	MC1 patients 2.1 point (20.4%) mean reduction versus placebo at 12 months
Urquhart^13^	2021	RCT protocol	CLBP with disc herniation *N* = 170, 85 per group	Amoxicillin clavulanate (Aspen Pharmacare Australia, NSW)	0.5 g Q12h	90 days	12 months (3, 6, 9 months secondary assessments)	The LBP NRS will be assessed	Not being assessed

Oral coamoxiclav was chosen for CLBP antimicrobial chemotherapy by Albert et al. in 2005, based on the advice of infectious disease physicians, and others followed the regimen^5^. Since then, a substantial body of work has emerged in response to the success of Albert’s studies, and it is a good time to consider this field and introduce perspectives from antibacterial pharmacokinetics and pharmacodynamics.

## Results

### Review of the literature

Coamoxiclav is approved for the treatment of acute bacterial otitis media, sinusitis, skin and skin structure, urinary tract, and bacterial infections of the lower respiratory tract, the latter being a major use. Exposure to antibiotic at the tissue site of infection is key to its efficacy, and exposure to amoxicillin has been estimated in target organs that are the sites of bacterial infection for approved indications. The relationship between tissue and serum concentrations has typically been used to summarize the penetration of antibiotics into the tissues. Oral coamoxiclav achieves high dose-dependent concentrations in the bronchial mucosa, for example, a single dose of amoxicillin/clavulanic acid (500 mg/125 mg or 500 mg/250 mg) achieved 7.2–10.1 µg/g of amoxicillin in the mucosa and 5.1–5.6 µg/ml in serum, indicating an accumulation of amoxicillin in the bronchial mucosa above serum concentration of 160%^2^. Intravenous (IV) administration of coamoxiclav (1000 mg/200 mg) achieved 20.8 µg/g of amoxicillin in the mucosa and 8.4 µg/ml in the serum; a tissue accumulation of 248%^2^. In contrast, the penetration of amoxicillin into the cortical hip bone was much lower after IV coamoxiclav (2000 mg/200 mg) with a mean bone C_max_ of ~16 µg/g^3^ to 26 µg/g^14^, or approximately 8.4% of the corresponding serum C_max_ ~190 µg/ml^3^.

There is only one report, by Housden and Sullivan, on the concentration of amoxicillin in herniated disc tissue after IV administration of coamoxiclav (1000 mg/200 mg) at the time of, or 60 minutes before general anaesthesia for lumbar discectomy to remove the attached herniated disc tissue^15^. The analytical method used to estimate antibiotic concentrations was not defined. The surgical procedure was estimated to take 30 min; therefore, the results provide an estimate of the attached herniated disc tissue amoxicillin at 30 and 90 min after IV administration. At both time points, one disc (20% of the samples) did not contain detectable levels of amoxicillin. In samples where amoxicillin was detected, the concentration ranged from 1.36 µg/g to 5.96 µg/g (4.4-fold) and 0.53 µg/g to 0.96 µg/g (1.8-fold) at 30 and 90 minutes, respectively (Table 2). The publication did not summarize the mean concentration of amoxicillin in herniated disc tissue, nor did it relate the tissue to serum concentrations. Intradiscal concentrations of clavulanic acid (Table 3) and cefuroxime (1.5 g, IV) were also reported^15^.

**Table 2:** Summary of intravenous amoxicillin serum and disc tissue exposure from Housden and Sullivan^15^.

	Amoxicillin concentration (µg/ml)				Percentage of disc/serum	
	Serum		Disc tissue			
Subject number	30 min.	90 min.	30 min.	90 min.	30 min.	90 min.
1	30.70		0.00		–	
2	46.70		3.81		8.16%	
3	60.90		3.51		5.76%	
4		5.60		0.53		9.46%
5		7.40		0.84		11.35%
6	50.67		5.96		11.76%	
7		19.03		0.60		3.15%
8		9.15		0.96		10.49%
9		5.09		0.00		–
10	37.13		1.36		3.66%	
Mean ± SD	45.22 ± 11.77	9.24 ± 5.70	2.93 ± 2.31	0.59 ± 0.37	7.33% ± 3.47	8.61% ± 3.72
Proportion of serum concentration in disc tissue			6.5%	6.4%	<7.3%	<8.6%

The proportion of serum amoxicillin concentration in disc tissue was calculated in two ways. The first used the mean concentrations at 30 and 90 min, and the second estimated the proportion for each subject and averaged the results. The latter did not account for tissue samples without detectable amoxicillin.

**Table 3:** Summary of intravenous clavulanic acid serum and disc tissue exposure from Housden and Sullivan^15^.

	Clavulanic acid concentration (µg/ml)				Percentage of disc/serum	
	Serum		Disc tissue			
Subject number	30 min.	90 min.	30 min.	90 min.	30 min.	90 min.
1	7.51		0.60		7.99%	
2	12.84		0.39		3.04%	
3	14.51		0.17		1.17%	
4		0.40		0.40		100%
5		1.18		0.43		36.44%
6	9.77		0.55		5.63%	
7		5.36		0.24		4.26%
8		2.80		0.40		14.28%
9		4.43		0.05		1.13%
10	6.70		0.46		6.86%	
Mean±SD	10.27 ± 1.50	2.87 ± 0.96	0.43 ± 0.07	0.30 ± 0.07	4.94%±1.25	31.22%±18.27
Proportion of serum concentration in disc tissue			4.2%	10.4%	4.94%	31.22%

The proportion of serum clavulanic acid concentration in disc tissue was calculated in two ways. The first used the mean concentrations at 30 and 90 min, and the second estimated the proportion for each subject and averaged the results.

Coamoxiclav is not indicated for the treatment of bone or disc infections. β-lactam antibiotics penetrate these tissues at concentrations <10% of those in serum. Pyogenic lumbar spondylodiscitis, a purulent bacterial infection, is typically treated with IV antibiotic administration for 4–6 weeks, followed by oral therapy for another 6 weeks^16^. *C. acnes* prosthetic joint infections are typically treated both surgically and medically, reducing the bacterial burden by synovectomy, prosthetic revision, and an initial IV antibiotic phase of 2–6 weeks followed by oral antibiotic therapy for another 6–10 weeks^17^. A case report on the treatment of *C. acnes* discitis associated with vertebral endplate changes described the use of ceftriaxone 2 g IV Q24 h × 6 weeks through a peripherally inserted central catheter^18^. The use of high-dose IV therapies with high serum exposure may lead to sufficient antibiotic exposure in the bone and disc tissues. Administration of intradiscal antibiotics twice weekly of 1–2 g cefazolin or vancomycin, depending on the bacterial infection for an average of 3.5 weeks, has also been reported^19^.

The standard use of prolonged IV antibiotics to treat pyogenic lumbar discitis raises the question of whether oral coamoxiclav achieves adequate tissue exposure to treat putative non-pyogenic disc infections. A comparison of tissue with serum concentrations or whether the tissue drug concentration is above the minimum inhibitory concentration (MIC) for the bacterial species of interest does not provide a robust assessment of whether adequate antibacterial exposure has been achieved. The in vivo pharmacokinetic/pharmacodynamic (PK/PD) target for exposure to amoxicillin, which correlates with the efficacy of its approved uses, is the achievement of a free antibiotic concentration in the target tissue above the MIC for more than 40% of the dosing interval; 40% fT>MIC^20,21^. Furthermore, an appropriate exposure test must be based on population assessment, with >90% of subjects achieving the appropriate PK/PD target, allowing for variable tissue exposure at the disc^20,21^. The efficacy of amoxicillin in treating disc infections can be limited by the low concentrations of amoxicillin in the disc, but this may be partially offset by the relatively sensitive C. *acnes* with a low MIC_90_ compared to other bacterial species, such as *S. aureus*. To assess whether the oral coamoxiclav dose regimens used to date provide adequate achievement of the PK/PD target in disc tissue, data from the literature were used to estimate the disc tissue exposure.

### Secondary pharmacokinetic analysis

In this study, the tabulated and graphical data in Housden and Sullivan (1993) were evaluated, and the mean amoxicillin concentrations in disc tissue after intravenous administration of coamoxiclav (1000 mg/200 mg) were estimated to be 2.9 µg/g at 30 min and 0.6 µg/g at 90 min, and the mean serum concentrations were 45.2 µg/ml and 9.2 µg/ml respectively, providing a disc/serum ratio of ~6.5% at both time points (Table 2)^15^. Intradiscal clavulanic acid penetration was also a fraction of the serum concentration, approximately 5–10%, with some variability at 90 min with low concentrations (Table 3).

**Fig. 1:**
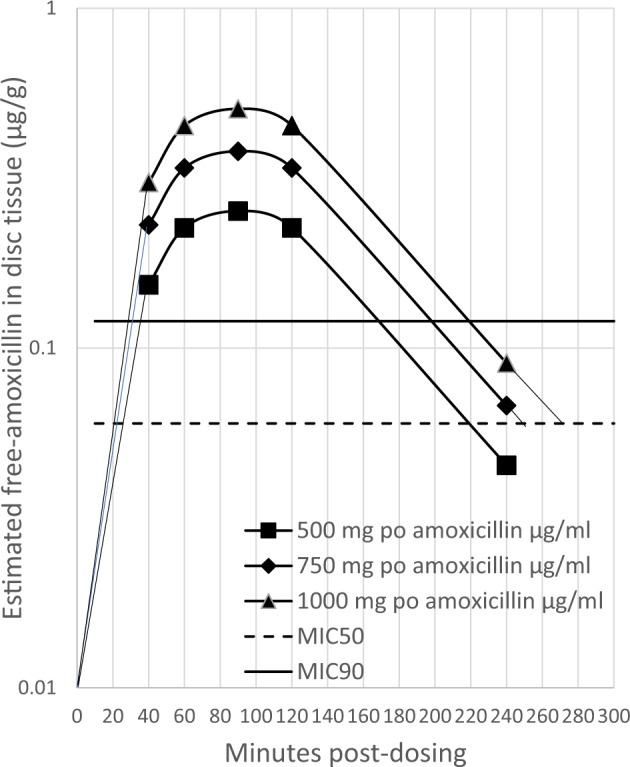
Estimate of free amoxicillin in herniated disc tissue. Horizontal lines at 0.06 µg/ml and 0.12 µg/ml free-amoxicillin serum concentrations of free amoxicillin are shown to represent MIC_50_ and MIC_90_ concentrations. Linear extrapolation was used to estimate the time above MIC_50_ and MIC_90_ values.

Assuming that exposure to amoxicillin in herniated disc tissue can also be estimated at 6.5% of the serum concentration after oral administration, and estimating the free amoxicillin concentration at 70% of the drug for disc tissue as it is for serum, and using linear proportional scaling for dose, the representative mean amoxicillin pharmacokinetics of herniated disc tissue was modelled (Fig. 1)^15,22,23^.

Estimation of the achievement of the PK/PD target of β-lactams requires knowledge of the MIC of the antibiotic against the bacterial species causing infection. The bacterial species most frequently isolated from the disc tissue of a patient with CLBP is *C. acnes*^1^. The amoxicillin concentration required to inhibit the in vitro growth of *C. acnes* strains derived from CLBP patient disc tissue is characterised by the MIC_50_ 0.06 µg/ml and the MIC_90_ 0.12 µg/ml, the concentrations of amoxicillin that inhibit 50% and 90% of strains, respectively^24,25^.

To estimate whether oral amoxicillin can reach the pharmacodynamic target in disc tissue, the duration of the amoxicillin concentration above the concentrations of MIC_50_ 0.06 µg/ml and MIC_90_ 0.12 µg/ml concentrations was estimated from Fig. 1 (Table 3). Oral amoxicillin dosing, Q12h, was unlikely to lead to effective exposure in herniated disc tissue, as twice-daily dosing failed to reach the 40% fT>MIC target (Tables 4 and 5). The mean exposure to 500 mg or 750 mg of Q8h oral amoxicillin may reach the efficacy target for 50% of strains but not 90% of strains, potentially limiting the efficacy of the treatment. The mean exposure to 1000 mg of oral amoxicillin Q8h may reach the target efficacy for 90% of strains (Tables 4 and 5).

**Table 4:** Estimated disc free-amoxicillin or free-cefuroxime concentration time above *C. acnes* MIC_50_ and MIC_90_.

	Oral dose of amoxicillin or cefuroxime time above *C. acnes* MIC_50_ and MIC_90_			
*C. acnes* MIC	250 mg	500 mg	750 mg	1,000 mg
Amoxicillin MIC_50_ 0.06 µg/ml	–	190 min	230 min	250 min
Amoxicillin MIC_90_ 0.12 µg/ml	–	130 min	170 min	190 min
Cefuroxime MIC_50_ 0.023 µg/ml	370 min	390 min	–	–
Cefuroxime MIC_90_ 0.047 µg/ml	245 min	315 min	–	–

Targets for 40% fT>MIC Q12h; 288 min, Q8h; 192 min, Q6h; 112 min.

**Table 5:** Estimated percentage of dosing interval fT>MIC for the mean exposure to free-amoxicillin or free-cefuroxime in disc tissue. Target is ≥40% for efficacy.

	MIC_50_ 0.06 µg/ml		MIC_90_ 0.12 µg/ml	
Oral dose of amoxicillin	Q12h	Q8h	Q12h	Q8h
500 mg	26%	40%	18%	27%
750 mg	32%	48%	24%	35%
1,000 mg	35%	53%	26%	40%
	MIC_50_ 0.023 µg/ml		MIC_90_ 0.047 µg/ml	
Oral cefuroxime dose	Q12h	Q8h	Q12h	Q8h
250 mg	51.4%	77.1%	34.0%	51.0%
500 mg	54.2%	81.2%	43.7%	65.6%

Inspection of the modelled herniated disc tissue exposure suggests that 8 to 12 h after dosing, when the next oral amoxicillin dose would be administered Q8h or Q12h, there would be little amoxicillin remaining in the disc and, therefore, little accumulation in the disc tissue after multiple doses (Fig. 1).

**Fig. 2:**
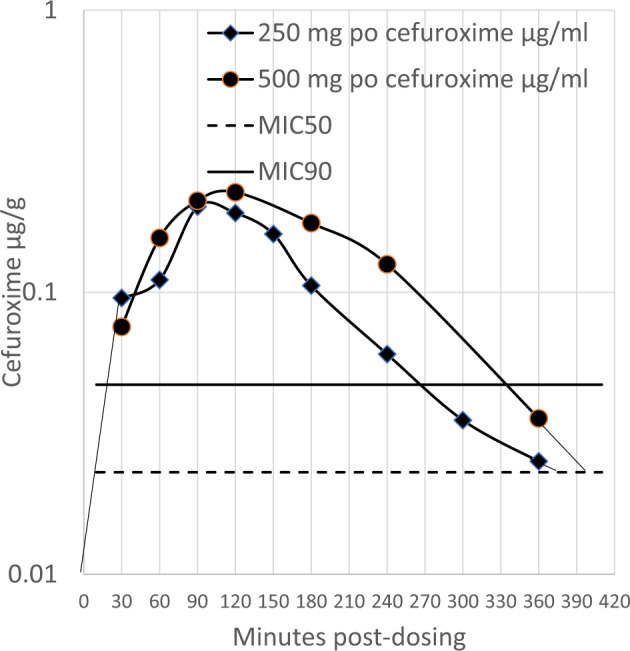
Estimate of free cefuroxime in nucleus pulposis disc tissue. Horizontal lines at serum concentrations of 0.023 µg/ml and 0.047 µg/ml free-cefuroxime serum concentrations are shown to represent the MIC_50_ and MIC_90_ concentrations. Linear extrapolation was used to estimate the time above MIC_50_ and MIC_90_ values.

Housden and Sullivan also reported cefuroxime concentrations in serum (mean 180.8 µg/ml) and attached disc tissue (mean 93.1 µg/g) after a 1.5 g IV dose, suggesting that disc tissue penetration achieved 51.5% of serum, substantially higher than amoxicillin penetration. However, other studies have not observed this high level of cefuroxime penetration into the disc, but they did not provide complete datasets^26,27^. Liang et al. provided a scatter plot of serum, annulus fibrosis, and nucleus pulposus concentrations, from which this study estimated the mean cefuroxime concentrations as 53.5 µg/ml, 6.1 µg/g and 3.9 µg/g respectively, and calculated the penetration of the nucleus pulposus cefuroxime at a serum concentration of 7.2%^27^. Gergs et al. provided a mean intradiscal cefuroxime concentration of 8.9 µg/g but only provided graphical serum data from which the C_max_ was estimated at 115 µg/ml, providing a penetration of 7.7% of serum into the disc tissue^26^. Using an average penetration of disc tissue from these two studies of 7.5%, oral cefuroxime axetil pharmacokinetic data for 250 mg and 500 mg doses and a free fraction of 0.67, a model of intradiscal exposure to cefuroxime was generated (Fig. 2)^28–32^.

To estimate whether oral cefuroxime axetil could reach the pharmacodynamic target in disc tissue, the duration of cefuroxime concentration above the cefuroxime concentrations of *C. acnes* MIC_50_ 0.023 µg/ml and MIC_90_ 0.0.047 µg/ml was estimated from Fig. 2 (Table 4)^33^. Oral cefuroxime axetil (500 mg Q12h) is likely to reach effective mean exposure to the disc, but a dose of 250 mg is not. Oral cefuroxime Q8h at 250 or 500 mg was likely to achieve mean efficacious exposure that reached the pharmacodynamic target for 90% of the strains (Table 5).

## Discussion

Clinical trials of oral coamoxiclav for up to 100 days to treat CLBP have shown significant benefits; however, the magnitude of clinical improvement has been variable^6,12,34^. Disc tissue is poorly vascularized and represents a challenging tissue in which to achieve adequate antimicrobial exposure^35^. Exposure to amoxicillin in herniated disc tissue is only 6.5% of the serum concentration, severely compromising the efficacy of oral amoxicillin in the treatment of bacterial disc infections compared to other sites of infection^15^. In similar studies, using HPLC to estimate antibiotic concentrations, other beta-lactams also penetrated degenerate disc tissue at serum concentrations of <10%; ceftriaxone, 7.7%^36^; cefuroxime, 7.5%^26,27^; but in healthy disc tissue cephadrine was not detected^37^. Differences in analytical methods might hamper detailed comparison. This study introduced antibacterial pharmacokinetic and pharmacodynamic approaches to assess whether oral coamoxiclav was likely to have been optimally dosed in CLBP trials to date.

Compared to IV amoxicillin administration, oral amoxicillin reaches a lower maximum serum concentration, and the time to the maximum serum concentration is delayed. Furthermore, at higher doses, a lower percentage of the dose is bioavailable, such that doubling the dose of 375 mg of amoxicillin leads to ~86% and quadrupling the dose to ~70% of the expected plasma concentration because of a putative capacity-limited carrier-mediated transport system^20,22,38^. At the time of the peak serum concentration in healthy fasting volunteers, a 500 mg oral amoxicillin dose reaches serum concentrations ranging from <1 µg/ml to ~18 µg/ml and depends on fasting/non-fasting status and the volume of water with which amoxicillin is administered^20,22,39,40^. Furthermore, the bioavailability of oral amoxicillin can be reduced by interactions with NSAIDs such as diclofenac, which is commonly used for pain management in patients with CLBP^41^.

This analysis suggested that oral Q12h doses of amoxicillin of up to 1000 mg are unlikely to reach antibacterial herniated disc tissue exposure. The 500 mg or 750 mg amoxicillin Q8h mean exposure may be effective for 50% of *C. acnes* strains, and the 1000 mg Q8h mean exposure may be effective for 90% of *C. acnes* strains. These were the mean exposures. Given that oral amoxicillin pharmacokinetics are variable, some disc tissues will at times contain no detectable amoxicillin, and that disc tissues evaluated to date may not reflect concentrations in the core of the nucleus pulposus, it is possible that all oral amoxicillin CLBP studies to date have been underdosed, and that the variability in terms of clinical effect is due to the wide variability in amoxicillin exposure at the site of infection. Disc amoxicillin is essentially eliminated during a single dose interval and it is not known if there is any accumulation in tissue concentration with multiple daily doses for up to 100 days. A wide range of amoxicillin exposures to disc tissue can be expected throughout the duration of up to 100 days of Q8h regimens, with a total of up to 300 doses. More than 1400 patients with CLBP were administered oral amoxicillin, with no apparent assessment of systemic or intradiscal pharmacokinetics (Table 1).

Antibiotic dose regimens should be selected based on population pharmacokinetics to provide a high probability of achieving the pharmacodynamic target and, therefore, a low risk of treatment failure (<10%) due to inadequate antibiotic exposure. Population pharmacokinetic analysis of oral amoxicillin based on serum concentration suggests that 500 mg of oral amoxicillin Q12h would be sufficient if amoxicillin was readily available in herniated disc tissue^20^. As amoxicillin poorly penetrates disc tissue, standard-dose regimens are inadequate for effective antimicrobial coverage in disc tissue. To date, the dose regimens tested in clinical trials to treat CLBP may have all suffered from a substantial proportion of treatment failures owing to inadequate exposure to disc tissue. Notably, no studies have explored dose escalation in terms of dose or dose interval, for example, >1 g of amoxicillin or administration four times a day.

The antibiotic sensitivity profile of *C. acnes* from skin or disc tissues suggests sensitivity to beta-lactams, for example, penicillin 94–100% susceptible^42–45^, benzylpenicillin 100%^46^, ampicillin 100%^47^, and amoxicillin 95.6-100%^25,43,48^. The coamoxiclav sensitivity of the few isolates that were insensitive to amoxicillin was not tested. The low frequency of isolates of *C. acnes* insensitive to amoxicillin suggests that coamoxiclav may not be necessary, as amoxicillin alone may be similarly effective, with the potential benefit of fewer adverse events related to the gastrointestinal tract^49^. Furthermore, low exposure of disc tissue to clavulanic acid can limit its activity against β-lactamases.

With such sparse data in the literature, a comprehensive pharmacokinetic and pharmacodynamic assessment of oral amoxicillin for the treatment of CLBP is not possible. However, this preliminary modelling provides insight and raises significant questions.

These findings have implications for the interpretation of efficacy for the low-dose group in Manniche et al. and the ongoing Urquart et al. studies that used Q12h dosing^8,13^ and may provide an explanation for the differences in results observed in the clinical trials of Albert et al. and Braten et al. ^6,12^. The higher dose of 1000 mg Q8h used in one arm of the study by Albert et al. versus 750 mg Q8h used by Braten et al. may have provided an advantage and may explain some of the observed differences in magnitude of clinical efficacy between the two studies.

Eight of the CLBP clinical studies used amoxicillin-clavulanate formulations, and one used amoxicillin alone (Table 1). Five studies disclosed the name of the antibiotic formulation used, while four did not. Amoxicillin and amoxicillin clavulanic acid formulations tend to be bioequivalent^50–53^. However, it should be noted that Braten et al. used an additional tablet encapsulation process to enable blinding of treatment^12^. Bioequivalence data on the encapsulated amoxicillin formulation are not provided, and it is not known whether encapsulation reduces oral bioavailability or C_max_ or alters Tmax, which could reduce exposure and efficacy.

This study had significant limitations. Primary intradiscal pharmacology studies all include few patients and provide a sparse dataset with few timepoints and replicates. Available data are on the IV amoxicillin concentration in herniated disc tissue that was attached to the disc and removed during surgery. Data on amoxicillin concentration in the axial nucleus pulposus after oral or intravenous administration are not available. The studies included subjects with CLBP and degenerate discs but did not assess whether they also had Modic changes in vertebrae adjacent to the disc which may alter antibiotic exposure. We assumed that the shape of the concentration-time curve within the disc mirrored that of the serum; however, this was based on the observation that the ratio was the same at only two-time points (Table 3). Currently, there are insufficient data to determine whether this assumption is valid for an entire dosing interval. The concentration of free amoxicillin within the disc matrix may differ from that in the serum. However, it is reasonable to expect that binding to the complex disc matrix may be higher than that to serum proteins and that at higher doses, amoxicillin serum concentrations may be lower than modelled because of the nonlinear bioavailability; therefore, this estimate of free amoxicillin in disc tissue in this study may be optimistic. The assumed concentration-time course used pharmacokinetics after a single administration because there are no data on the pharmacology of amoxicillin during 300 doses; therefore, antibiotic accumulation cannot be excluded. Substantial inter-patient and intra-patient variability of the bioavailability of amoxicillin leading to wide variation in serum concentrations may lead to substantial variability in intradiscal exposures during the extended period of dosing.

There is a continuing debate about the efficacy of oral amoxicillin in treating patients with CLBP and Modic Type 1 changes based on two large RCTs^6,12^. The first study by Albert et al., using 500 mg and 1000 mg of Q8h amoxicillin, demonstrated a substantial benefit of oral coamoxiclav in patients with CLBP and Modic type 1 changes, with an indication of dose response^6^. Second, a similar study, but with multiple significant differences, using 750 mg of over-encapsulated Q8h amoxicillin, showed a significant antibiotic benefit for subjects with Modic change type 1. Subsequent post hoc analysis indicated that a subgroup of subjects with large vertebral Modic lesions responded to antibiotic therapy, with a substantial reduction in disability^12,34^. It is not clear whether these subjects were more likely to have a bacterial infection of their discs or whether large vertebral Modic oedema allowed greater exposure to amoxicillin through the disc endplates, or both.

Perhaps, the debate should focus on the selection of the antibiotic and dosage regimen. The publications using oral amoxicillin to treat CLBP by Braten et al. and the AIM study group and their subsequent analyses should be considered with caution^12,34,54,55^. The differences between Albert et al. and Braten et al. oral amoxicillin RCTs may be at least partially explained by both studies being in a steep part of the amoxicillin dose-response at the intervertebral disc. Some patients respond to oral antibiotics; however, their use must be optimized^6,34^. Oral cefuroxime axetil may be an alternative to amoxicillin, as it is more potent against *C. acnes* and the modelling presented here suggests that it may provide a better chance of adequate intradiscal exposure at a lower dose.

A planned Cochrane review on the use of oral amoxicillin to treat CLBP with Modic changes, based on the RCT studies summarized here, may be premature and may have a greater impact when diverse antibiotics and regimens have been evaluated and optimized and further RCTs conducted^56^.

This review identified gaps of knowledge to set priorities for further research. Additional high-quality studies with well-validated analytical methodology such as Liang et al.,^27^ investigating the intradiscal pharmacokinetics of oral antibiotics in subjects with Modic type 1 and Modic type 2 changes are required to inform the optimisation of dosing, and future studies should at least incorporate an assessment of serum/plasma antibiotic concentrations at timepoints during the 100 days of administration. It is recommended that expertise in antibacterial pharmacokinetics and pharmacodynamics should be integrated into the design and execution of future studies.

## Methods

### Review of the literature

One author (LC) provided the primary review of the literature. The time period was not restricted. Publications in English with abstracts were inspected to identify those relevant to this study. PubMed listed similar articles, and reference lists were inspected for relevant articles.

The literature was searched through PubMed between 13-15 September 2022 with the following keywords and search terms; ((amoxicillin OR clavulanic OR clavulanate OR amoxiclav OR bioclavid OR augmentin) (lumbar OR vetebral OR spine) (disc OR disk OR pulposus OR herniation OR herniated) (CLBP or ‘Lower Back Pain’ OR Low Back Pain’ OR sciatica OR discectomy)) 31 results; ((antibiotic OR teicoplanin OR ceftriaxone OR ertapenem OR Vancomycin OR Ciprofloxacin OR Clindamycin OR doxycycline OR quinolone) (lumbar OR vertebral OR spine) (disc OR disk OR pulposus OR herniation OR herniated) (CLBP or “Lower Back Pain” OR “Low Back Pain” OR Sciatica OR discectomy)) 243 results; (amoxicillin bioequivalence) 71 results; (amoxicillin (lung OR sinus OR ear OR skin OR urinary OR tissue) (concentration OR penetration OR distribution)) 997 results; (acnes antibiotic resistance) 464 results; (amoxicillin drug interactions) 630 results; (Cefuroxime Pharmacokinetics) 511 results. Furthermore, the pharmacology literature Web resource https://pkpdai.com/pkdocsearch was used with the search terms amoxicillin, bioclavid, amoxiclav, and clavulanate^57^.

### Secondary pharmacokinetic analysis

This study extends the analysis provided by Housden and Sullivan^15^ by introducing an additional analysis based on PK/PD principles. An estimate of the achievement of the β-lactam antibiotic PK/PD target (40% fT>MIC) in the disc tissue was made from data from the literature. The oral amoxicillin (250 mg) serum time-concentration curve of Spyker et al. was used to provide representative data (Table 1^22^). Estimates of serum amoxicillin concentrations at time points for 500, 750, and 1000 mg doses were obtained by multiplying the 250 mg dose data by 2x, 3x, or 4x, respectively. The nonlinear absorption of amoxicillin can overestimate its concentration at higher doses by up to 30%^20,32,38^. The penetration of disc tissue to 6.5% of the serum concentration (see below) and the use of a 70% free fraction allowed the estimation of free amoxicillin in disc tissue over time after oral dosing^15,23^. The elimination of amoxicillin follows a simple exponential curve, which is a straight line on a logarithmic plot over a wide range of concentrations^58^. This justified a straight-line extrapolation of the time-concentration curve. The duration above the *C. acnes* amoxicillin MIC_50_ or MIC_90_ concentration was estimated and expressed as a fraction of the dosing interval duration^24,25^. In the Housden and Sullivan study, the concentration of cefuroxime in herniated disc tissue was also reported^15^. A literature search identified two additional studies reporting the intradiscal concentrations of cefuroxime^26,27^. Neither of the studies presented complete datasets. Estimates of tissue cefuroxime concentrations from the graphs allowed the calculation of the approximate ratios of serum to disc tissue concentrations. Cefuroxime is more potent than amoxicillin against *C. acnes* with an MIC_90_ of 0.047 µg/ml vs. 0.12 µg/ml respectively^24,25,33^.

## Data Availability

The data used for the secondary pharmacokinetic analysis was derived from publications as described in methods.

## Code availability

Not applicable as no custom code was used.
